# Computer-aided clinical image analysis for non-invasive assessment of tumor thickness in cutaneous melanoma

**DOI:** 10.1186/s13104-021-05650-4

**Published:** 2021-06-14

**Authors:** Marios Papadakis, Alexandros Paschos, Andreas Manios, Percy Lehmann, Georgios Manios, Hubert Zirngibl

**Affiliations:** 1 Division of Surgery II, University of Witten-Herdecke, Heusnerstr. 40, 42283 Wuppertal, Germany; 2 Department of Dermatology, Helios St. Elisabeth Hospital Oberhausen, Oberhausen, Germany; 3 Department of Surgical Oncology, School of Medicine, University Hospital Heraklion, Heraklion, Greece; 4 Department of Dermatology, Helios University Hospital, Wuppertal, Germany; 5 Department of Computer Science and Biomedical Informatics, University of Thessaly, Volos, Greece; 6 Department of Surgery, Helios University Hospital, Wuppertal, Germany

## Abstract

**Objective:**

Computerized clinical image analysis is shown to improve diagnostic accuracy for cutaneous melanoma but its effectiveness in preoperative assessment of melanoma thickness has not been studied. The aim of this study, is to explore how melanoma thickness correlates with computer-assisted objectively obtained color and geometric variables. All patients diagnosed with cutaneous melanoma with available clinical images prior to tumor excision were included in the study. All images underwent digital processing with an automated non-commercial software. The software provided measurements for geometrical variables, i.e., overall lesion surface, maximum diameter, perimeter, circularity, eccentricity, mean radius, as well as for color variables, i.e., range, standard deviation, coefficient of variation and skewness in the red, green, and blue color space.

**Results:**

One hundred fifty-six lesions were included in the final analysis. The mean tumor thickness was 1.84 mm (range 0.2–25). Melanoma thickness was strongly correlated with overall surface area, maximum diameter, perimeter and mean lesion radius. Thickness was moderately correlated with eccentricity, green color and blue color. We conclude that geometrical and color parameters, as objectively extracted by computer-aided clinical image processing, may correlate with tumor thickness in patients with cutaneous melanoma. However, these correlations are not strong enough to reliably predict tumor thickness.

## Introduction

Tumor thickness at the time of surgical treatment remains the most widely accepted and accurate predictor of prognosis in patients with cutaneous melanoma. It also defines the size of the surgical margin and helps determine which patients should undergo sentile lymph node biopsy. For these reasons, much effort is being directed at obtaining reliable information regarding tumor thickness prior to the operation, as thick tumors have to be excised with a larger surgical margin.

Several tools have been implemented to support these efforts. Dermoscopy has become very popular the last 2 decades. It is not invasive and is proven to facilitate melanoma diagnosis of clinically suspicious pigmented lesions. However, its effectiveness as a thickness assessment tool is controversial. Some authors found specific dermatoscopic patterns, such as pigment networks, gray-blue areas and vascular patterns to be associated with thick lesions [1] or blue white veils, milky-red areas and shiny-white streaks to be associated with ulceration and mitotic rate [2], whereas other could not confirm these findings [3]. Several models to predict tumor thickness out of dermoscopical images have been proposed, but all require high expertise, as they are based in presence of absence of particular patterns. Interestingly, Pizzichetta et al. report that dermoscopical characteristics do not differ between patients with in situ melanoma and patients with invasive melanoma [4]. Finally, most dermoscopic criteria were described in the context of superficial spreading melanoma [5].

Other novel non-invasive methods, such as high-frequency sonography and epiluminescence light microscopy show promising results, but, apart from their cost, they also require training and are observer-dependent.

Computerized clinical image analysis is also shown to improve diagnostic accuracy for cutaneous melanoma [6]. It is non-invasive, cheap and obviates interpretative problems as it is based on mathematic analysis. Several color and geometric parameters are found to be associated with melanoma. However, to the best of our knowledge, a digital image processing analysis to demonstrate correlation of specific color and geometric variables with melanoma thickness has not been reported. The aim of this study is to explore how melanoma thickness correlates with computer-assisted objectively obtained color and geometric variables.

## Main text

### Materials–methods

#### Patient recruitment, image collection, storage and image database

Following the study approval by the institutional Ethics Committee of University Witten/Herdecke, we retrospectively reviewed and analyzed the computerized medical records of all patients, diagnosed with cutaneous melanoma in a tertiary university hospital in Germany during a 3-year period. The study included patients with histologically confirmed melanomas with available clinical images shot with a digital camera along with a ruler. Exclusion criteria included patients younger than 18 years old, in-situ melanomas, ulceration, extensive regression as reported in the pathologist report, mucosal tumors, acral melanomas and amelanotic lesions. All lesions were photographed at admission with the same commercial digital camera at the same high-resolution (1600×1200 pixels) and with a ruler being aligned beside the lesion to allow for correct scaling of the images. All photos were obtained from the same educated nurse to minimize inconsistencies in methodology. Any hairs on the lesion were removed with a razor prior to photography. The photos were then uploaded to a local server in the highest-quality jpeg format. After obtaining informed written patient consent, the lesions were excised under local anaesthesia and the diagnosis was histologically confirmed. Melanoma thickness was considered the one reported at the pathologist report.

#### Image processing

All color images obtained, underwent digital processing with an automated non-commercial software developed from one of the authors (AM) for study purposes. The software provided measurements for geometrical variables, i.e., overall lesion surface, maximum diameter, perimeter, circularity, eccentricity, mean radius, as well as for color variables, i.e., range, standard deviation, coefficient of variation and skewness in the red, green, and blue (RGB) color space. Each RGB component pixel was assigned an intensity value between 0 and 255 (8-bit accuracy). All 15 variables studied are explained in Table 1 [7].

**Table 1:** Explanation of geometric and color variables studied

Classification	Parameter	Explanation
Geometry variables	Area	Lesion surface area, measured in cm^2^
	Maximum diameter (MaxD)	the longest line that joins two points on the border of the lesion, measured in cm
	Perimeter	Total boundary length of the region of interest (i.e., lesion), measured in cm
	Circularity	Ratio of the perimeter of the lesion divided by the perimeter of a circle with the same midpoint and same area as the lesion
	Mean radius (Rm)	Mean value of the lesion’s radii
	Eccentricity	Distance between color and geometric midpoint within the lesion
Color variables (values 0–255)	Range of red, green, blue	Range of values of red, green, blue intensity
	Mean red, green, blue	Mean value of red, green, blue intensity within the lesion
	SD of red, green, blue	Standard deviation of red, green, blue intensity within the lesion
	CV of red, green, blue	Expresses the standard deviation of red, green, blue intensity values as mean percentage
	Skewness from Gaussian curve (red, green, blue)	Deviation of each color’s histogram from the normal distribution curve

#### Statistical analysis

Normal distribution was determined using histogram plots, box plots and the Shapiro–Wilk test. Continuous data were normal or approximately normal distributed and are, therefore, presented in mean–standard deviation form. Correlations between the measured parameters and the melanoma thickness were calculated using the Pearson correlation test at the 95% significance level. Correlations were considered strong at r ≥ 0.6, moderate at 0.2 ≤ r < 0.6 and weak at r < 0.2. Data analyses were performed using SPSS 23.

## Results

One hundred fifty-six lesions from an equal number of patients were included in the final analysis. The mean tumor thickness was 1.84 mm (range 0.2–25). Melanoma thickness was strongly correlated with overall surface area, maximum diameter, perimeter and mean lesion radius. Thickness was moderately correlated with eccentricity. Regarding color parameters, only moderate and weak correlations were observed. Tumor thickness was moderately correlated with green color (standard deviation (SD), coefficient of variation (CV), range) and blue color (SD, range). Statistically significant weak correlations were observed between melanoma thickness and the values of the mean red, the skewness of green and the CV of the blue intensity. All correlations are presented in Table 2.

**Table 2:** Color and geometrical variables of melanoma patients, as extracted with digital image processing

Variable	Mean (SD)	r coefficient	p value
Geometric variables			
Area (cm^2^)	2.5 (3.7)	0.72	**0.000**
MaxD (cm)	1.9 (1)	0.65	**0.000**
Perimeter (cm)	5.5 (2.9)	0.62	**0.000**
Circularity (ratio)	1.1 (0.07)	− 0.05	0.56
Rm (cm)	0.8 (0.4)	0.65	**0.000**
Eccentricity (cm)	0.03 (0.02)	0.46	**0.000**
Color variables			
Mean red	156 (32)	− 0.17	**0.04**
SD red	33 (11)	0.11	0.19
CV red	23 (11)	0.14	0.09
Range red	191 (35)	0.15	0.06
Skewness red	− 0. 3 (0.7)	0.16	0.05
Mean green	97 (24)	− 0.17	**0.03**
SD green	30 (6)	0.28	**0.000**
CV green	33 (12)	0.33	**0.000**
Range green	194 (32)	0.23	**0.003**
Skewness green	0.43 (0.57)	0.18	**0.03**
Mean blue	93 (25)	− 0.04	0.64
SD blue	30 (6)	0.21	**0.01**
CV blue	35 (12)	0.18	**0.02**
Range blue	202 (33)	0.21	**0.009**
Skewness blue	0.47 (0.54)	0.08	0.31

All continuous variables are approximately normally distributed and therefore expressed in mean-deviation form. Statistical significance: p-value < 0.05

## Discussion

Tumor thickness remains the most important prognostic factor in patients with cutaneous melanoma, with 1 mm being the common cut-off value between thin and thick tumors. The latter require broader excision and further investigation with sentinel lymph node biopsy. Despite this fact, correct thickness assessment can be a difficult task, as even highly experienced dermatologists can correctly classify only about 90% of the cases [8].

Several studies analyzed digital dermoscopy images to determine color and geometrical parameters observed in thin and thick lesions [4, 8, 9]. Clinical image analysis is considered a tool with high diagnostic accuracy for melanoma [6], but its effectiveness in non-invasive assessment of the tumor thickness has not been studied. Our findings demonstrate that specific geometrical and color parameters correlate with melanoma thickness, the correlations, though, are not strong enough to reliably predict tumor thickness.

The lesion diameter is part of the ABCDE criteria and represents the only criterion that can be studied objectively and thus with higher reliability. Digital image analysis provides measurements with the skin under tension, which can differ from these of the ex vivo specimens provided from the pathologist, due to tissue shrinkage up to 20%, especially at patients younger than 50 years old [10]. Digital image processing prevents thus inter-observer variability. We found that the largest lesion diameter correlates strong with the tumor thickness. Seidenari et al. also observed this relationship at lesions located on the trunk and limbs, but not at head melanomas, attributing this finding to the large portion of head lesions developing in situ and persisting as lentigo maligna with long lasting horizontal growth [10]. Argenziano et al. found this correlation being statistically significant only in the case of thick lesions, but their interpretation was based on ordinal rather than continuous data (i.e., diameter was classified in categories) [1].

In our study, thickness was moderately correlated with eccentricity. Eccentricity is considered a special case of asymmetry. According to Lorentzen et al.: “*with progression of the tumour, the cancerous tissue will be found not only at the periphery but will also cause bulk asymmetry, displacing the tissue with a less abnormal appearance*” [3]. Therefore, eccentric lesions are possible thicker.

Melanomas greater than 1 mm thick are reported to have a larger area and a greater presence of blue [8]. Our study confirms this finding, as the overall area as well as the range and the standard deviation of the blue color was found to increase with thickness (strong and moderate correlation, respectively). The same study also reports a greater randomness in the disposition of pattern components in melanomas with thickness > 1 mm, as shown by the presence of red, green and blue multicomponent patterns. According to the authors, such patterns express the number, dimensions and differences between objects color within the lesion and are correlated with the structural asymmetry and ‘disorder’ inside the lesion. However, a detailed description and definition of what should be considered a multicomponent pattern is not provided. In our analysis, we preferred to assess the color distribution in terms of color range for all three color intensities, rather than color percentiles inside the lesions.

There are different approaches regarding the assessment of color features. Several authors suggest assessment of all six colors that a pigmented lesion can present: black, dark brown, light, brown, blue-gray, red, and white [11]. This classification is common in dermoscopy, since the different colors are believed to represent different melanin locations: melanin appears black when it is located in the stratum corneum or upper epidermis, brown in the deep epidermis and gray or blue in the dermis. Red is associated with dilation of blood vessels and white with regression and/or scaring. Adopting this classification, one has to segment each lesion into their constituting colors [11]. We avoided this approach, as it is not fully objective and, moreover, is difficult to set the threshold of the covered lesion area for the color to be counted.

Argenziano et al. reported that the presence of blue-black color, i.e., a combination of blue and black pigmented areas involving at least 10% of the lesion surface, is associated with the presence of nodular melanoma, i.e., high-thickness melanoma. However, the assessment was based on qualitative observer-dependent evaluations, not on quantitative parameters [5].

We conclude that geometrical and color parameters, as objectively extracted by computer-aided clinical image processing, may correlate with tumor thickness in patients with cutaneous melanoma. However, these correlations are not strong enough to reliably predict tumor thickness.

## Limitations

Our study has some limitations that should be considered. Firstly, it is a single-centre retrospective study and its results cannot be easily generalized. Secondly, we included only melanoma patients with available clinical images, made before the diagnosis was established. This could represent a selection bias, as patients with large abnormal pigmented lesions are more likely to seek medical advice. Similarly, particularly suspect cases were more likely to be photographed and therefore recruited. Thirdly, as the specimens were analyzed from different pathologists during the study period, the possibility of interpretation bias of tumors’ thickness cannot be ruled out, especially regarding regression. Last but not least, our analysis considered only the clinical bidimensional parameters of the lesion.

## Data Availability

The datasets used and/or analysed during the current study are available from the corresponding author on reasonable request.

## Abbreviations

RG: Bred, green, and blue color space
SD: Standard deviation
CV: Coefficient of variation
